# How Differential Dimensions of Social Media Overload Influences Young People’s Fatigue and Negative Coping during Prolonged COVID-19 Pandemic? Insights from a Technostress Perspective

**DOI:** 10.3390/healthcare11010006

**Published:** 2022-12-20

**Authors:** Hua Pang, Min Ji, Xiang Hu

**Affiliations:** 1 School of New Media and Communication, Tianjin University, Tianjin 300072, China; 2 School of Political Science and Public Administration, Henan Normal University, Xinxiang 453007, China; 3 Department of Psychology, University of Constance, 78464 Constance, Germany

**Keywords:** social networking sites, social media overload, fatigue, negative coping, young people

## Abstract

Although social networking sites have emerged as the primary source of information for young people, there is a dearth of knowledge concerning the underlying associations between differential aspects of social media overload and whether social media overload ultimately influenced people’s negative coping strategies during the prolonged COVID-19 pandemic. In order to fill this gap in existing knowledge, the current research employed the stressor–strain–outcome (SSO) theoretical paradigm to explicate social media fatigue and negative coping strategies from a technostress perspective. The study used cross-sectional methodology, whereby 618 valid questionnaire responses were gathered from WeChat users to assess the conceptual model. The obtained outcomes demonstrated that information overload and communication overload positively impacted young people’s fatigue. Furthermore, these two patterns of perceived overload heighten social media fatigue, which ultimately leads to young people’s negative coping with the COVID-19 pandemic. These findings would extend the present social media fatigue and technical stress literature by identifying the value of the SSO theoretical approach in interpreting young people’s negative coping phenomena in the post-pandemic time.

## 1. Introduction

In recent years, social networking sites (SNS) have increasingly permeated into our everyday lives and have triggered considerable academic attention [1,2,3,4]. In particular, during the prolonged COVID-19 pandemic, SNS were the efficient and effective channels for individuals to acquire online psychosocial assistance and pandemic-related information [5,6]. Prior research has demonstrated that SNS utilization during this global health crisis exerted the potential to impact users’ health and psychosocial state, particularly among young people [7,8,9]. Whilst the adoption of SNS technologies could have some advantages to individuals in a global pandemic, researchers also underline the adverse side of SNS and assert that the negative impacts of SNS adoption during the global public health event remain sufficiently unearthed [10,11]. Only a few attempts have uncovered the underlying impact of social media services on the younger generations’ sense of fatigue and negative coping strategies during the prolonged global pandemic. Under this particular circumstance, the current work probes the potential influence of social media overload on young people’s sense of fatigue and negative coping with stressors based on the information and technical characteristics of this new technology.

The majority of previous studies have discovered that the initial stage of lockdown resulted in increased SNS adoption among the younger generation, and that, following this period, their passion for various social media platforms began to decrease; this was reflected in the SNS usage decline during the later period of the pandemic shutdown [2,8,12]. For instance, a significant amount of social media users were deviating from engagement on popular SNS such as Facebook, YouTube, and Myspace because of suffering from fatigue [6,13]. WeChat, one of the most successful and leading SNS provider in mainland China, is inevitably confronted with the same challenge [14,15]. Recently, most WeChat users have encountered adverse emotions about this new technology because of boredom and tiredness with the huge quantities of useless content and superfluous requirements from online friends for “forwarding,” “liking,” as well as “leaving comments.” Noticeably, in studies of this new phenomenon, some researchers from all over the world have carried out quantitative investigations to identify the potential antecedents and outcomes of social media fatigue [16,17].

Although recent studies have asserted that some determinants of social media fatigue may be produced by physiological and behavioral stress-related circumstances, including perceived overload, feelings of dissatisfaction or regret, and social communication engagement [4,5,7,18], little research has explored the possible influence of distinct patterns of social media overload on users’ social media fatigue. Moreover, on a personal level, social media fatigue may lead to a deterioration in a person’s emotional or psychosocial strengths, whereby social media users are prone to unhealthy conduct [19,20,21]. To date, there is limited knowledge concerning how the distinct dimensions of social media overload (system dimension, information dimension, and communication dimension) as vital stressors would result in young people’s negative coping behaviors. Furthermore, prior studies have claimed that social media fatigue could directly result in users abandoning their continuous intentions and behaviors [13,22]. However, it is unclear if social media fatigue might serve as a moderator in the association between social media overload and negative coping strategies during the global health crisis. Considering that social media are widely utilized, worldwide, it is necessary to examine their psychosocial influence in relation to social media fatigue and coping with stressors.

To fill the aforementioned research void, this work uses the stressor–strain–outcome (SSO) theoretical framework to uncover the associations between differential compositions of social media overload and whether these distinct patterns of overload could lead to negative coping strategies. In particular, this current study examines three distinct types of social media overload (system overload, information overload, and communication overload) rather than merely treating social media overload as one single stressor. In addition, this article investigates the underlying associations between different patterns of overload, social media fatigue and negative coping, which might offer a better understanding of the vital role of social media overload in interpreting young people’s negative coping styles. Furthermore, in this work, a research model is proposed to identify the factors that significantly impact on individuals’ negative coping in the WeChat context, with a particular concentration on the mediation roles of social media fatigue. Thus, this study addresses the significant research gap concerning users’ inner psychological fatigue involved in SNS adoption and offers fresh light on factors that could affect their coping with stress in the period of the pandemic. Furthermore, the theoretical framework adds to the present literature by uncovering the dissimilar influences of perceived overload on individuals’ fatigue and negative coping, as well as the mediating role of fatigue from the technostress perspective.

## 2. Theoretical Background and Hypotheses Formulation

### 2.1. Stressor–Strain–Outcome (SSO) Theoretical Paradigm

Proposed by some scholars, the SSO theoretical paradigm was adopted in psychosocial studies to interpret the negative influence of related technostress on the behavior of users via psychological strain [23,24]. According to this perspective, stressors have direct influence on individuals’ strain factors, which subsequently contribute to behavioral outcomes [2,25]. To be specific, the concept stressor reflects the environmental variables that trigger stress and impact people’s psychological conditions. Strain could be conceptualized as psychological consequences caused by stress variables [20,24]. The outcomes indicate that individuals’ personal responses to existing strain factors and avoidance or oppositional behaviors are recognized as corresponding consequences [10,11]. In earlier studies, the underlying principles of the SSO paradigm have been extensively utilized to understand job-related stressors in typical workforces or physical commercial circumstances [26,27]. In the era of the swift development of social media technology, the overuse of communication technologies, social comparation, and social overload are stressor variables affecting young people’s emotions and practices (strain, such as emotional tiredness, depression, or unsatisfaction) towards SNS, which ultimately result in diverse negative psychological or physiological consequences, consisting of declined academic achievement or users’ discontinuous usage behaviors.

On the basis of previous studies, the SSO research model was chosen as the overarching theoretical paradigm to investigate young people’s psychological mechanisms and negative responses during this coronavirus pandemic [24,28]. As displayed in the above-mentioned literature, the main purpose behind selecting the SSO paradigm over other frameworks is that the SSO paradigm is exactly in line with the primary objective of this current research, i.e., to uncover which factors would initiate social media fatigue and what are its potential outcomes and consequences from the individual-level user perspective [16,24]. Although SSO theory has been extensively utilized for probing stress-related circumstances and subsequent consequences within the context of the application of new communication technology [17,28], very little research has systematically unpacked the external environmental situations that increase the likelihood of social media exhaustion translating into negative coping behavior.

Additionally, many researchers have claimed that the application of the SSO theoretical paradigm framework could offer deeper interpretations, which are vital for comprehending the antecedents and consequences of negative behaviors [10,26,29]. Further, using the SSO framework is beneficial as it makes a distinction between general psychological strain and related behavioral consequences, instead of treating different psychological and behavioral consequences as an overall concept of strain [16,27]. Therefore, considering the unique circumstances of the worldwide pandemic and the crucial function of the technological characteristic of SNS, the SSO model provides a suitable framework for probing the adverse influence of social media overload on young people’s psychological processes and negative coping strategies.

### 2.2. The Association between Perceived Overload and Social Media Fatigue

The adoption of SNS could exert positive consequences, such as increasing a person’s supportive resources, improving their psychological state, as well as facilitating civic involvement [12,20,30]. Prior research has also highlighted that the proliferation of the Internet and SNS leads to some harmful outcomes, such as depression, mental health issues, as well as decreased performance when users experience social media overload [6,13]. The phenomenon of social media overload can be described as users being exposed to vast quantities of content and interaction needs through social media that might demand inner energy and psychological processing beyond their abilities [26,31,32]. Additionally, previous investigations have theorized and empirically demonstrated the different dimensions of perceived overload [16,20]. One study of the ICT-productivity paradox identified three essential components of technology overload; namely, information dimension, communication dimension, and system feature dimension [33]. Information overload arises when the quantity and complexity of the data that must be stored and processed overwhelms an individual’s information processing capacity [12]. System feature overload occurs when a communication service is so complicated for specific tasks and the addition of diverse novel characteristics is outweighed by the potential influence of technical sources and complicacy of usage [29]. Communication overload indicates the undesirable circumstance that arises when interaction needs from various platforms, such as SNS, exceed people’s communication ability. In the online context, various patterns of overload could be correlated with individual’s social media fatigue from using SNS [8,13]. However, at present, there is little research examining how various types of overloads induce psychological changes that result in social media users’ fatigue. Accordingly, this article concentrates on information overload, system feature overload, as well as communication overload, which are widespread in the context of social media.

As social media fatigue is receiving considerable close attention, an accumulating body of research on the topic has been implemented in multiple disciplinary fields, such as health psychology, medical science, and communication domains [6,17,34]. Social media fatigue refers to a unified, subjective feeling of weariness triggered by the additive communications of both physiological and psychological factors [29,35]. Thereby, this term underlines the adverse perceptions of feelings experienced by users of SNS, such as perceived stressor, social anxiety, boredom and depressive illnesses. Recently, some scholars have begun to probe the antecedents and consequences of social media fatigue. Prior studies have identified that overloads of technology, information, or communication may cause social media fatigue via individuals’ engagement and communication on diverse social media services [12,36]. More specifically, information overload could predict users’ fatigue on the social media platform. The possible reason for this is that SNS users’ capabilities for handling and processing information could not catch up with the speed of information accumulation on SNS [20,31]. In sum, the explosive growth and rapid spread of digital information could result in information overload, which negatively impacts individuals’ psychology and behavior.

Additionally, based on cognitive load theory, people’s cognitive overload can arise if the quantity of cognitive resources required to finish certain tasks outweighs the quantity of resources that are saved within the cognitive systems [16,29,37]. In the social media context, system feature overload encompasses the growth of the equipment and processes that would impose psychological and physical constraints on individuals. Previous research reveals that system feature overload has become a stressor for people in computer-mediated environments [30,35]. When there are constant transformations within system characteristics and capabilities are particularly sophisticated for individuals, system feature overload may occur and result in detrimental outcomes such as fatigue [30,38]. Therefore, under the setting of WeChat, young people use the platform in a number of ways, and various properties are provided for diverse aims, which would cause people to become bored of using SNS and experience fatigue [3,5]. Furthermore, communication overload could also cause a sense of fatigue and even lead to severe mental and physical problems [8,28]. In fact, the high-speed and convenience of communication through SNS have been optimized by the renewed systems, which enable people to stay in touch and access vast quantities of posts and messages. Users of SNS who receive an excessive amount of contact may feel overloaded, which will exacerbate social media fatigue. Consequently, the following three hypotheses are proposed:

**Hypothesis 1 (H1).**  *Information overload positively influences social media fatigue.*

**Hypothesis 2 (H2).**  *System feature overload positively influences social media fatigue.*

**Hypothesis 3 (H3).**  *Communication overload positively influences social media fatigue.*

### 2.3. The Association between Social Media Fatigue and Negative Coping

When suffering from social media fatigue, people normally generate multidimensional psychological reactions associated with SNS services, such as social anxiety, dissatisfaction, and depressive symptoms [29,30,39]. Under these circumstances, users with such adverse emotions are more inclined to adopt a series of negative coping tactics to avoid feelings of discomfort [12,17]. The concept of coping strategies refers to certain conscious behaviors, both behavioral and psychological, that people use to master, tolerate, decline, or alleviate stressful occasions, to handle personal or interpersonal problems [30,40]. That is, these are related strategies and countermeasures that people may turn to in order to manage either external or internal requirements that develop pressure and handle displeasurable emotion. Negative coping strategies include thoughts and actions that divert attention away from the source of stress, such as isolation and distraction [5]. Typically, coping behavior could be classified into positive coping and negative coping [39]. Positive coping indicates taking a direct and reasonable approach to settle an issue. By comparison, negative coping reflects handling matters through avoidance, secede or denial [10,41]. Negative coping primarily includes asocial avoidant behaviors that are not concentrated on the stressors, such as diversion, or withdrawal thinking [5,39].

Previous studies have demonstrated that negative coping is typically related to social media users’ unhealthy emotional states and tends to increase their deviant behaviors, such as dissatisfaction or aggression [5,29]. Individuals’ physical and psychological strength have been shown to deteriorate as a result of social media fatigue [26]. Thus, people who experience weariness are more prone to alter their current circumstances and worsen their mental well-being [10,12]. Under such circumstances, individuals usually take a couple of measures to minimize the harmful outcomes of being overwhelmed by SNS adoption. A recent survey demonstrated that social network fatigue could influence social media users’ distraction and withdrawal thinking [31]. In addition, the perspective of perceived overload revealed that individuals’ social media fatigue and unhappiness may result in individuals’ discontinuous usage [11].

During the pandemic period, negative coping strategies tended to manifest through maintaining personal emotions, avoiding COVID-19 relevant information and refusing to contact others. In this extreme circumstances, people decreased their social media fatigue, generated from perceived overload, by decreasing the frequency of use or entirely forsake private SNS accounts in order to separate themselves from negative sentiments associated with SNS [6,12,30]. In addition, negative coping could not transform such devastating and threatening conditions but only help people feel comfortable provisionally [38,42]. Owing to the preponderance of COVID-19-related information on social media, utilizing negative coping strategies would hinder individuals’ adaption to the global crisis [12,42]. A handful of previous studies have suggested that negative coping has become a dangerous element for depressive moods and social anxiety [25,38,39]. Accordingly, the work anticipates that negative coping is a result of fatigue and probes the indirect influence of perceived overload on negative coping through social media fatigue during this COVID-19 pandemic. Only understanding these potential effects can people better govern social media fatigue and refrain from negative coping. The following hypothesis is thus proposed:

**Hypothesis 4 (H4).**  *Social media fatigue positively influences negative coping.*

### 2.4. The Association between Social Media Overload and Negative Coping

Previous studies have claimed that the social media ecosystem contains pressures that cause people to feel internal tension, which finally results in preventable behavioral consequences of social media use [27,43]. Scholars of psychology have suggested that psychological fatigue would cause lower engagement and diminished learning performance, impair self-regulation behaviors, and exert detrimental influences on individuals’ emotional reactions [2,11,36]. In the context of social media, a number of empirical studies have investigated the connection between users’ perceptions of being overwhelmed and the outcomes that follow that perception. For instance, Lee et al. (2016) highlighted that greater perceptions of different overloads can result in fatigue and feelings of stress and fatigue will ultimately influence individuals to discontinuance or even withdraw completely [29]. Yao and Cao verified that users’ behavioral reactions, such as brief suspension, use control, and usage termination, are strongly connected with the stresses generated by SNS [20]. Recent research reveals that, depending on the degree of fatigue, cognitive overload and privacy invasion results in fatigue, which may have several negative consequences, such as users’ feelings of fatigue and strain [11].

In the midst of the unpredictability of the worldwide pandemic, the overwhelming presence of COVID-19 information on a variety of social networking sites has induced adverse emotional reactions. On the one hand, the quantity of information, pictures, and other web-based messages published or reposted on SNS are rising dramatically, but the capability to deal with this information is limited [25,26]. On the other hand, users of SNS were confronted with great volumes of daily information, resulting in their tiredness and health anxiety [10]. Thus, the dark sides of information overload consist of people’s inability to obtain reliable content and the stress of a limited amount of time and the negative psychological effects [38]. Additionally, in order to meet users’ psychological needs, mobile social media developers and administrators often work to enhance functionality and service capabilities by introducing new technological affordances. Despite several novel functions of SNS productions and consumptions being more unique and efficient, an excess of functions in these platforms would make users feel boredom, overwhelmed and experience a decline in interest [20]. In particular, the features of WeChat have expanded from earlier message dissemination to business and public health applications with add-on features such as lifestyle display, online consultations, and virtual transaction [44]. In the digital era, one person’s inability to handle brand-new technologies or the outcomes of utilizing these technologies would lead to psychological health issues.

Furthermore, social media was recognized as a virtual web-based community, and thus communication overload could appear in the network environments. When people’s social networks demand superfluous energy and effort to sustain interpersonal relations, these changes may cause serious mental or physical diseases such as pressure tension and tiredness. As a direct result of the enormous growth of SNS, individuals have to cope with unwanted human relationships and excessive interaction from these platforms, social media users may feel overwhelmed and fatigue as to they cannot effectively handle such situations [1,29]. In the context of WeChat, due to the excessively large number of weak and strong ties, users typically simply browse information from their circle of friends without actively engaging in communication; this may result in greater health anxious and fatigue. Based on the above-mentioned studies, this study anticipated that social media users’ perceived information overload, system feature overload and communication overload are positively associated with negative coping. The study thus proposes these following three hypotheses:

**Hypothesis 5 (H5).**  *Information overload positively influences negative coping.*

**Hypothesis 6 (H6).**  *System feature overload positively influences negative coping.*

**Hypothesis 7 (H7).**  *Communication overload positively influences negative coping.*

## 3. Study Model and Methodology

### 3.1. Study Model

**Figure 1:**
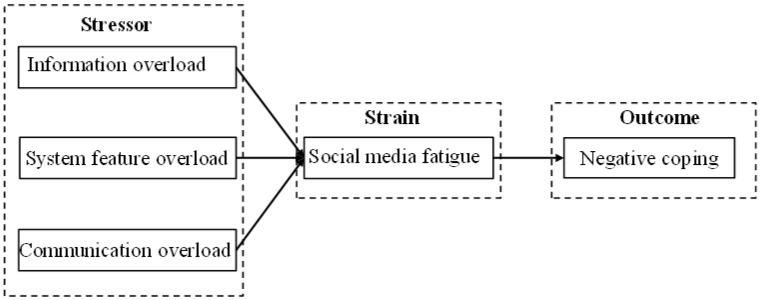
The proposed conceptual research model.

To empirically interpret social media fatigue, this study utilizes the stressor-strain-outcome study framework. According to this classic framework, the study systematically explores the potential linkages between distinct dimensions of social media overload (i.e., information overload, system feature overload and communication overload), social media fatigue and negative coping among young people. Specifically, the study theorizes that people’s perceived information overload, system feature overload and communication overload are significantly associated with increased fatigue, which ultimately leads to higher levels of negative coping. Figure 1 displays this proposed theoretical model and the corresponding hypotheses.

### 3.2. Participants and Procedure

The questionnaires were gathered from two universities in mainland China via a web-based survey, between 30 September and 30 October 2022. The research recruited university students as target participants in this survey, as they constitute the largest population of SNS members and represent the most vibrant consumers in the era of SNS [2,3]. Therefore, the research survey’s subjects were aged between 18 and 29. A total of 679 subjects were gathered in the study. After eliminating 61 replies due to unreliability, a valid sample of 618 respondents was used.

### 3.3. Construct Measurement

The scales are presented in the Appendix A.

#### 3.3.1. Information Overload

The measurement for information overload is derived from earlier investigations [12]. Examples of statements are “I am always distracted by excessive amount of COVID-19-related information on WeChat” and “I have to deal with too much COVID-19-related information every day that I get from WeChat.” Using a 5-point Likert scale ranging between strongly disagree (1) and strongly agree (5), the participants were required to respond according to their agreement or disagreement with these items, based on the previous months. The score was ultimately averaged to form an index of information overload (Cronbach’s alpha = 0.85, M = 3.81, SD = 0.52).

#### 3.3.2. System Feature Overload

The measure for system feature overload was derived from Lee et al. [29]. Examples of items are “I’m frequently distracted by WeChat features unrelated to my core aim” and “WeChat is helpful by providing elements that make social performance more difficult.” The scale contains five statements, and each statement is assessed between 1 = strongly disagree and 5 = strongly agree, with a higher score indicating greater system feature overload (Cronbach’s alpha = 0.86, M = 3.69, SD = 0.61).

#### 3.3.3. Communication Overload

The communication overload assessment scale was modified from earlier research [5,29]. The representative constructs are “I get too many messages from friends or acquaintances through WeChat” and “I receive too many WeChat alerts when doing other things.” The students were asked to respond according to their feelings or thoughts over the past month. A Likert scale from 1 (strongly disagree) to 5 (strongly agree) was used for all of the questions. The higher the score, the more perceived communication overload the respondents experienced (Cronbach’s alpha = 0.78, M = 3.88, SD = 0.51).

#### 3.3.4. Social Media Fatigue

This research adopted three statements from prior studies to assess the university students’ social media fatigue [12,43]. Examples of items are “I feel frustrated when using WeChat these days” and “I feel emotionally drained after using WeChat these days.” Using a scale of 1 to 5, with 1 being strongly disagree and 5 being strongly agree, the students were required to report if they agree or disagree with these constructs. The score was then averaged to create an index of social media fatigue (Cronbach’s alpha = 0.91, M = 3.88, SD = 0.64).

#### 3.3.5. Negative Coping

The construct of negative coping is measured with three statements borrowed from the study of Zhang et al. [5]. The representative construct is “Today, I wish I could alter what occurred or how I felt.” According to a 5-point scale ranging between 1 (strongly disagree) and 5 (strongly agree), the students were required to report to what extent they agree or disagree with the above statements. These statements were subsequently averaged to stablish an index of negative coping; Cronbach’s alpha = 0.95 M = 3.23, SD = 0.78).

#### 3.3.6. Socio-Demographic Variables

This study included the primary socio-demographic characteristics of age, gender, education level, and WeChat usage experience serving as the control variable.

## 4. Data Evaluation

Initially, in order to obtain a fresh insight into the demographic characteristics and WeChat adoption statistics of young people, we carried out basic descriptive statistics studies. The zero-order correlations method was then used to assess any possible associations between the key constructs. Ultimately, the proposed study model was examined using the structural equation modeling (SEM) technique. The technique was employed to evaluate the psychometric qualities of every latent variable assessment as well as the direction of relationships among the constructs and corresponding significance level. In general, all of the current data analyses were implemented by using IBM SPSS 25.0 and IBM AMOS 24.0.

## 5. Study Results

### 5.1. Descriptive Statistics

Before exploring the survey questions, we carried out preliminary analyses to obtain the summary of descriptive statistics about the survey sample. The descriptive analyses discovered that, among the 618 participants, 51.6% of them were males. The ages of the respondents ranged between 18 and 32, with half of them being between the ages of 24 and 32 (SD = 0.86). In terms of degree level, the majority of the participants were well-educated: 70.6% had an undergraduate degree and 20.2% had a Master’s degree. For WeChat usage experience, 81.4% have used WeChat for over 4 years. Additionally, the descriptive analysis also shows that approximately 76.7% of the subjects spent more than two hours on WeChat daily and 81.7.9% of them had more than 200 friends on WeChat. The descriptive data for all of the respondents are shown in Table 1.

### 5.2. Zero-Order Correlation between Research Variables

Table 2 displays the zero-order correlation between the research measures. Zero-order correlations were carried out to uncover how these scaled variables are related. Among the hypothetical overload factors, information overload (r = 0.329, *p* < 0.01), system feature overload (r = 0.254; *p* < 0.05), and communication overload (r = 0.256; *p* < 0.01) were positively correlated with social media fatigue. The results demonstrated that younger generations who experienced greater perceived overload about the COVID-19 pandemic reported more emotional fatigue. Social media fatigue was positively correlated with negative coping (r = 0.483; *p* < 0.01). Furthermore, negative coping was positively related to information overload (r = 0.334, *p* < 0.01), system feature overload (r = 0.343; *p* < 0.01), and communication overload (r = 0.366; *p* < 0.01). Thus, the different dimensions of social media overload would have significant correlations with the negative coping outcomes.

### 5.3. Assessment of Path Model

The structural equation modeling technique (SEM) was utilized to gauge the proposed research model. The total fit indices for this hypothetical framework are excellent as the outcomes are within the acceptable boundaries: chi-square/df is 1.387, RMSEA = 0.014, RMR = 0.010, CFI = 0.991, AGFI = 0.916, IFI = 0.982, TLI = 0.913. Then, we evaluated this structural research model to explore the hypothesized associations. The results showed that most of the standardized route coefficients were statistically significant, providing evidence for the study hypotheses. Information overload (b = 0.230, *p* < 0.001) and communication overload (b = 0.149, *p* < 0.05) were found to exert a significant and positive effect on fatigue, suggesting they are vital predictors of fatigue. Therefore, hypotheses 1 and 3 are statistically corroborated. Moreover, social media fatigue (b = 0.379, *p* < 0.001) significantly influences negative coping, displaying that it is the determinant of negative coping. Hence, hypothesis 4 is statistically supported.

**Figure 2:**
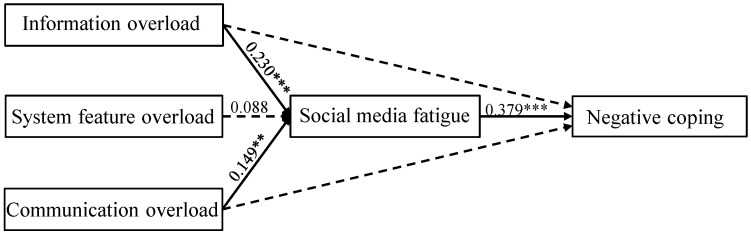
The model testing result. Note. ** *p* < 0.01, *** *p* < 0.001.

Contrary to expectations, the participants’ perceived system feature overload (β = 0.088, *p* = 0.100) indicated an insignificant influence on social media fatigue. Furthermore, the SSO framework underlines the indirect influence of stressors on corresponding outcomes. Accordingly, mediation analysis was implemented to gauge whether the impact of information overload and communication overload on negative coping could be mediated by social media fatigue. To be specific, the impact of information overload on the individuals’ sense of fatigue becomes insignificant (*p* < 0.05) when social media fatigue (mediator variable) intervenes between these two related constructs. Similarly, the indirect path between communication overload and social media fatigue through social media fatigue is also insignificant. Consequently, the results confirm that social media fatigue may mediate the impact of information overload and communication overload on negative coping. Thus, hypotheses 5 and 7 are supported by the statistical evidence. Figure 2 illustrates the results of the hypotheses testing and path coefficients.

## 6. Discussion

### 6.1. Summary of the Main Results

Applying the S-S-O theoretical framework, the present article is an initial study contributing to the existing body of research on comprehending the dark consequences of young people’s WeChat usage amid the COVID-19 pandemic in mainland China. More specifically, the article explores the detrimental impacts of differential dimensions of social media overload of WeChat on young people’s sense of fatigue and, subsequently, the impact of social media fatigue on their negative coping. Furthermore, the moderating impact of social media fatigue on the association between social media overload and negative coping is also assessed.

Firstly, the findings demonstrate that information overload is significantly associated with the exhaustion brought on by social media fatigue. The results imply that the perceived abundance of COVID-19 information on WeChat might contribute to the increased levels of social media fatigue among young people. Given that the younger generation were exposed to a plethora of COVID-19-related material on WeChat amid the global health crisis, such information overload would trigger their mental discomfort, thereby resulting in a sense of fatigue. The outcome is in line with prior investigations and emphasizes the negative influences of information overload on WeChat users’ psychological state under the COVID-19 situation [2,10,12]. Contrary to our expectations, system feature overload could not significantly associate with social media fatigue. One possible reason for this is that the development of social media technologies might not bring about cognitive and emotional stress for young people, which would not lead to mental discomfort. Another possible reason is that individuals could use the different functions selectively and overlook some functions that are not required and does not directly create fatigue [1,6].

Secondly, this study finds that communication overload is positively related to social media fatigue. This result is consistent with previous studies, suggesting that communication overload exerts a significant and positive impact on the development of perceived fatigue [26,29]. Owing to the advancement of social media usage, WeChat has evolved into a multi-functional ecosystem consisting of official accounts, Online payment, Little Programs, as well as other communication functions [45,46]. The increasing number of connections and the growth of one’s social circle cause WeChat users to have to cope with excessive interaction requirements from social networks. In particular, lots of WeChat users report that, despite the tremendous expansion of their WeChat buddy lists, the vast majority of their contacts remain strangers [11,47]. They would experience social media fatigue if the demands of managing their social networks and interactions exceeded their capacity for involvement.

Thirdly, the findings suggest that social media fatigue could significantly increase young people’s negative coping. The outcomes of this study support the previous studies indicating that users’ negative emotional states might be brought on by social media fatigue [3,10,43]. This finding could potentially be in line with earlier research, which indicated that social media fatigue is normally deemed as the situational factor affecting negative reactions of individuals [4,7]. In addition, social media fatigue may act as a moderator in the detrimental impact of information and communication overload on users’ negative coping, according to the mediation study. This again emphasizes the mediating function of strain between stresses and unfavorable outcomes among people in the setting of social media.

### 6.2. Theoretical and Practical Implications

Several important theoretical contributions may be drawn from this investigation. Firstly, the results of this paper provide novel insights into the mediator role of social media fatigue in the association between the distinct dimension of overload and negative coping strategies in the setting of WeChat. In particular, this study may add to the body of prior research in the areas of the effect of system features, information, and communication overload, as well as the empirical confirmation of their correlations with social media fatigue and negative reactions. Secondly, the research could advance the existing comprehension of negative coping strategies by adopting the classic SSO paradigm and explaining the psychosocial drivers of negative coping among young people. Thirdly, the study could expand the detailed understanding of social media fatigue in the WeChat context. On the basis of the existing studies, this article presents a novel research perspective, which strives to make up for the shortages of previous studies merely concentrating on the perspective of overload. Future research could also use Online Photovoice (OPV) to conduct research on the same or similar topics, i.e., using OPV to explore the role of social media among young people and understanding how social media use impacted youth’s coping mechanisms during the pandemic, as OPV gives opportunities to the participants to express their own experience with as little manipulation as possible, if any, compared to traditional quantitative methods [48].

Practically, this research offers some vital insights for SNS designers, marketers, healthcare providers, and health educators. The results could assist social media designers to comprehend the causes of fatigue on the social media platforms and thereby declining the negative reactions. Specifically, designers or developers of SNS may alleviate individuals’ sense of social media fatigue by adjusting the speed of system updates, optimizing use functions, and allowing users to optionally utilize the functions and services based on their personal demands. Additionally, social media designers could decrease the dissemination of unnecessary information and supervise advertising push services. Social media marketers could also decrease people’s feeling of fatigue according to the results. Furthermore, in order to prevent an overwhelming volume of communication through SNS, designers could develop certain programs to help users control the number of friends on the platform. Healthcare providers could adopt strategies to assist users in dealing with stresses. For example, they may offer users a more regulated environment in which they can restrict contact requests at certain times and apply optional filters to information that they do not find especially interesting. Meanwhile, health educators may provide users with the ability to temporarily turn off real-time push notification or instant messaging functionality.

### 6.3. Limitations and Potential Research Directions

There are also several research limitations in this study. First, as the survey data were gathered from one country and from young people, under 32 years old, it is probable that the present results would alter if alternative age groups and national backgrounds were considered. Although the younger generation accounts for the vast majority of WeChat users, it might be better for following studies to verify the research model with a wider age spectrum across distinct countries to improve the robustness of the obtained outcomes. Second, the current article employs a cross-sectional design and gathered data from a web-based questionnaire; this procedure may produce methodological biases. For instance, some social media overload constructs are highly connected. As a result, when they were included in the same model, there may be a multicollinearity problem. That might explain why the link between system feature overload and social media tiredness was not statistically significant. Moreover, SEM did not provide ways to test the significance of the indirect paths. Future studies could use Hayes macro based on bootstrap approaches. To address this limitation, subsequent studies could employ a longitudinal approach and qualitative methodologies to further comprehend the influence of social media overload on young people’s WeChat fatigue and negative reaction during the global pandemic. Third, the article merely pays attention to the potential influence of the perception of overload on negative coping and investigates the mediating role of social media fatigue. However, some studies have indicated that self-disclosure, social media stalking, social comparisons, and other variables could also lead to users’ fatigue and negative response. Hence, future research could probe the indirect influence of overload on negative behaviors through various factors.

## 7. Closing Conclusions

Drawing upon the stressor-strain-outcome framework, this study develops a conceptual model to discover the impact of information, system feature and communication overload on social media fatigue, and to further uncover whether these factors induce negative coping. These findings suggest that perceived overload (i.e., information overload, system feature overload and communication overload) directly leads to negative coping in the WeChat context. Additionally, social media fatigue could play a mediating role in the association between social media overload and negative coping. As a result, this research goes one step further by analyzing both the direct and indirect effects of various patterns of social media overload. The new understandings derived from the article would provide conceptual connections for perceived overload that were experimentally confirmed in terms of social media fatigue and corresponding responses.

## Figures and Tables

**Table 1:** Demographic statistics of respondents (N = 618).

Respondents	Category	Count	Percentage
Gender	Male	319	51.6
	Female	299	48.4
Age	18–20	42	6.8
	21–23	261	42.2
	24–26	249	40.3
	27–29	56	9.1
	30–32	10	1.6
Education background	High school or below	12	1.9
	Undergraduatedegree	436	70.6
	Postgraduate degree	162	26.2
	Doctoral degree	8	1.3
WeChat using experience	<1 year	1	0.2
	1–2 years	15	2.4
	2–3 years	32	5.2
	3–4 years	67	10.8
	>4 years	503	81.4
Daily duration of WeChat use	<1 h	20	3.2
	1–2 h	124	20.1
	2–3 h	177	28.6
	3–4 h	142	23.0
	>4 h	155	25.1
Number of WeChat friends	<100	33	5.3
	101–200	80	12.9
	201–300	115	18.6
	301–400	113	18.3
	>400	277	44.8

**Table 2:** Zero-order correlations among key variables (N = 618).

Key Variables	1	2	3	4	5
1. Information overload	1				
2. System feature overload	0.537 **	1			
3. Communication overload	0.459 **	0.386 **	1		
4. Social media fatigue	0.329 **	0.254 *	0.256 **	1	
5. Negative coping	0.334 **	0.343 **	0.366 **	0.483 **	1

Note. * *p* < 0.05, ** *p* < 0.01.

## Data Availability

The data of this study are available from the corresponding author upon reasonable request.

## Appendix A. Measurements

Information overload (IFO)
IFO1: I am always distracted by excessive amount of COVID-19-related information on WeChat.
IFO2: I have to deal with too much COVID-19-related information every day that I get from WeChat.
IFO3: I’m having trouble synthesizing too much COVID-19-related information on WeChat.
System feature overload (SFO)
SFO1: I’m frequently distracted by WeChat features unrelated to my core aim.
SFO2: WeChat is helpful by providing elements that make social performance more difficult.
SFO3: The WeChat features I utilize are sometimes more complicated than their duties.
Communication overload (CMO):
CMO1: I get too many messages from friends or acquaintances through WeChat
CMO2: I send more messages to friends than I wish to on WeChat.
CMO3: I receive too many WeChat alerts when doing other things.
CMO4: I always feel overloaded with communication from WeChat.
CMO5: I get more communication messages and news than I can handle on WeChat.
Social Media Fatigue (SMF):
SMF1: I feel frustrated when using WeChat these days.
SMF2: I feel emotionally drained after using WeChat these days.
SMF3: I feel irritable after using WeChat for hours these days.
Negative Coping (NGC):
NGC1: Today, I wish I could alter what occurred or how I felt.
NGC2: Today, I fantasized about a better time or location than the one I was in.
NGC3: I had thoughts or hopes about how things would turn out today.
